# Supplementary material for: Pediatric Inflammatory Multisystem Syndrome (PIMS) Did Occur in Poland during Months with Low COVID-19 Prevalence, Preliminary Results of a Nationwide Register
Source: J Clin Med. 2020 Oct 22;9(11):3386. doi: 10.3390/jcm9113386 (PMC7690437; doi:10.3390/jcm9113386)
Supplement: Supplementary file 1 [file jcm-09-03386-s001.pdf]

Table S1. Demographic and Clinical Characteristics of the Patients, According to Age Group and SARS-CoV-2 Status.

**Characteristics**	**Overall (*N* = 39)**	**<5 years (*N* = 25)**	**≥ 5 years (*N* = 14)**	***p*-Value**	**SARS-CoV-2 History Positive (*N* = 9)**	**SARS-CoV-2 History Unknown (*N* = 18)**	**SARS-CoV-2 History and Results Negative (*N* = 12)**	***p*-Value**
	**n, n/N or med (% or IQR)**	**n, n/N or med (% or IQR)**	**n, n/N or med (% or IQR)**		**n, n/N or med (% or IQR)**	**n, n/N or med (% or IQR)**	**n, n/N or med (% or IQR)**	
Age [years]	3.1 (1.4–6.6)	1.8 (0.85–2.8)	9.15 (6.4–12.15)	**<0.01***	10.5 (7–13.4)	3.3 (1.9–5.4)	1.2 (0.5–1.6)	**<0.01***
Male sex	29 (74%)	21 (84%)	8 (57%)	0.42	4 (44%)	13 (72%)	12 (100%)	**0.047***
Caucasian race	39 (100%)	25 (100%)	14 (100%)	-	9 (100%)	1 (100%)	12 (100%)	-
BMI [kg/m²]	15.7 (14.6–17.4)	15.8 (14.5–17.3)	15.4 (14.6–19.1)	0.74	15.8 (15.3–19.1)	15.6 (14.4–16.5)	15.6 (14.5–18.1)	0.48
Comorbidities								
Any	6/39 (15%)	3/25 (12%)	3/14 (21%)	0.37	2/9 (22%)	3/18 (17%)	1/12 (8%)	0.62
Acquired immunodeficiency	4/39 (10%)	3/25 (12%)	1/14 (7%)	0.54	1/9 (11%)	2/18 (11%)	1/12 (8%)	0.96
Chronic heart disease	2/39 (5%)	2/25 (8%)	0/14	0.41	0/9	1/18 (6%)	1/12 (8%)	0.87
Obesity	2/36 (6%)	0/23	2/13 (15%)	0.15	1/9 (11%)	1/16 (6%)	0/11	0.91
Clinical features								
Temperature [°C]	39.0 (39.0–40.0)	39.0 (39.0–40.0)	39.0 (39.0-40.0)	-	40.0 (39.0–40.0)	39.5 (39.0–40.0)	39.0 (39.0–40.0)	-
Length of fever [days]	8 (6.0–10.5)	8 (6.0–10.0)	6.5 (6.0–12.5)	0.76	6 (6.0–11.0)	8 (6.0–12.25)	8 (6.75–9.0)	0.61
Any dermatologic	37 (94%)	20 (80%)	13 (93%)	0.39	7 (78%)	17 (94%)	11 (97%)	0.39
Rash	34 (87%)	22 (88%)	12 (86%)	0.60	6 (67%)	17 (94%)	11 (97%)	0.11
Hands and feet erythema or swelling	23 (59%)	13 (52%)	10 (71%)	0.20	7 (78%)	11 (61%)	5 (42%)	0.24
Peeling	17 (46%)	10 (40%)	6 (44%)	0.87	3 (33%)	10 (56%)	4 (33%)	0.34
BCG injection site erythema	1 (3%)	1 (4%)	0	0.76	0	1 (6%)	0	-
Any mucocutaneous	30 (77%)	23 (92%)	10 (71%)	0.11	5 (56%)	15 (83%)	10 (83%)	0.22
Conjunctivitis	26 (67%)	17 (68%)	9 (64%)	0.54	4 (44%)	13 (72%)	9 (75%)	0.27
Mucosal changes	27 (69%)	18 (72%)	9 (64%)	0.43	4 (44%)	15 (83%)	8 (67%)	0.12
Lymphadenopathy	20 (51%)	15 (60%)	5 (36%)	0.13	3 (33%)	9 (50%)	8 (67%)	0.32
Any musculoskeletal	15 (39%)	6 (24%)	9 (64%)	**0.01***	5 (56%)	6 (33%)	4 (33%)	0.48
Arthritis (swollen joints)	8 (21%)	2 (8%)	6 (43%)	**0.01***	4 (44%)	3 (17%)	1 (8%)	0.11
Arthralgia (without swelling)	9 (23%)	2 (8%)	7 (50%)	**<0.01***	3 (33%)	5 (28%)	1 (8%)	0.33
Myalgia	7 (18%)	3 (12%)	4 (29%)	0.19	3 (33%)	1 (6%)	3 (25%)	0.15
Any gastrointestinal	24 (62%)	13 (52%)	11 (79%)	0.09	7 (78%)	10 (56%)	7 (56%)	0.52
Nausea or vomiting	15 (40%)	6 (24%)	9 (64%)	**0.02***	5 (56%)	6 (33%)	4 (33%)	0.48
Abdominal pain	16 (42%)	6 (24%)	10 (71%)	**<0.01***	5 (56%)	7 (39%)	4 (33%)	0.22
Diarrhea	13 (33%)	8 (32%)	5 (36%)	0.54	5 (56%)	4 (22%)	4 (33%)	0.22
Any neurologic	30 (77%)	21 (84%)	10 (71%)	0.29	5 (56%)	13 (72%)	11 (92%)	0.16
Neck stiffness	3 (8%)	2 (8%)	1 (7%)	0.71	1 (11%)	0	2 (17%)	0.61
Somnolence	17 (44%)	12 (48%)	5 (36%)	0.34	4 (44%)	6 (33%)	7 (58%)	0.39
Headache	6 (15%)	0	6 (43%)	**0.001***	5 (56%)	1 (6%)	0	**<0.01***
Seizures	1 (3%)	0	1 (7%)	-	1 (11%)	0	0	-
Anosmia or aguesia	1 (3%)	0	1 (7%)	-	1 (11%)	0	0	-
Partial paralysis	1 (3%)	0	1 (7%)	-	1 (11%)	0	0	-
Cutaneous hyperalgesia	8 (21%)	6 (24%)	2 (14%)	0.38	1 (11%)	3 (17%)	4 (33%)	0.39
Irritability	20 (51%)	15 (60%)	5 (36%)	0.13	3 (33%)	10 (56%)	7 (58%)	0.46
Photophobia	1 (3%)	1 (4%)	0	-	0	1 (6%)	0	-
Any respiratory	16 (41%)	14 (56%)	4 (29%)	0.09	4 (44%)	5 (28%)	7 (58%)	0.24

Cough	6 (15%)	4 (16%)	2 (14%)	0.63	2 (22%)	2 (11%)	2 (17%)	0.74
Chest pain	1 (3%)	0	1 (7%)	-	1 (11%)	0	0	-
Dyspnea	4 (10%)	2 (8%)	2 (14%)	0.45	2 (22%)	0	2 (17%)	0.42
Coryza	6 (15%)	6 (24%)	0	0.05	0	1 (6%)	5 (42%)	**0.03***
Sore throat	8 (21%)	7 (28%)	1 (7%)	0.12	1 (11%)	3 (17%)	4 (33%)	0.39

BCG: Bacillus Calmette–Guérin vaccine; BMI: body mass index; IQR: interquartile range, med: median.
*statistically significant ($p < 0.05$)

Table S2. Laboratory Results, According to Age Group and SARS-CoV-2 Status.

**Characteristics**[a]		**Overall (*N* = 39)**	**< 5 years (*N* = 25)**	**≥ 5 years (*N* = 14)**	***p*-Value**	**SARS-CoV-2 History Positive (*N* = 9)**	**SARS-CoV-2 History Unknown (*N* = 18)**	**SARS-CoV-2 History and Results Negative (*N* = 12)**	***p*-Value**
		**med (IQR)**	**med (IQR)**	**med (IQR)**		**med (IQR)**	**med (IQR)**	**med (IQR)**	
Neutrophil count [×10^9/L]		10.8 (7.5–15.1)	10.1 (7.5–14.6)	11.1 (7.1–19.4)	0.51	13.5 (9.3–20.0)	9.8 (6.4–12.6)	10.8 (9.8–14.7)	0.49
Hemoglobin [g/dL]		9.7 (8.3–10.6)	9.4 (8.1–10.6)	10.0 (9.7–10.6)	0.19	9.8 (7.8–10.6)	10.2 (9.6–10.9)	8.65 (7.8–9.6)	0.11
Procalcitonin [ng/dL]		1.0 (0.3–4.0)	1.1 (0.3–2.0)	1.0 (0.4–14.9)	0.64	9.3 (0.5–18.0)	1.0 (0.4–1.9)	0.8 (0.3–12.4)	0.37
ESR [mm/hr]		63.0 42.5–93.5	70.0 45.0–92.0	54.0 29.5–89.75	0.37	61.0 43.5–93.5	44.5 23.0–71.0	83.5 58.5–107.5	0.12
BNP [pg/mL]		295.4 (89.3–868.8)	269.0 (68.3–321.0)	568.1 (102.0–1764.0)	0.18	768.6 (126.7–2054.3)	321.0 (269.0–456.0)	147.0 (60.0–295.7)	0.07
Troponin [ng/L]		4.0 (2.5–24.6)	3.2 (2.3–16.5)	11.3 (2.9-109.2)	0.24	16.6 (4.7–153.4)	2.9 (2.5–11.6)	3.2 (2.2–13.6)	0.19
Fibrinogen [mg/dL]	Min	309.5 (232.0–391.5)	313.0 (255.5–353.5)	304.0 (198.0–471.0)	0.81	198.0 (53.2–270.0)	313.0 (273.0–369.0)	297.0 (230.0–349.0)	0.63
	Max	567.0 (501.0–650.0)	567.0 (391.0–693.0)	588.0 (506.3–648.5)	0.91	596.0 (567.0–648.0)	571.0 (368.5–661.5)	564.0 (530.0–567.0)	0.71
INR		1.3 (1.1–1.4)	1.3 (1.1–1.3)	1.3 (1.2–1.5)	0.37	1.4 (1.2–1.5)	1.3 (1.1–1.3)	1.2 (1.1–1.2)	0.42
LDH [U/L]		269.0 (232.0–393.0)	274.5 (244.8–443.8)	244.0 (212.0–307.0)	0.29	232.0 (160.0–307.0)	278.0 (244.5–694.5)	263.0 (244.0–341.0)	0.38

BNP: brain natriuretic peptide; ESR: erythrocyte sedimentation rate; INR: international normalized ratio; IQR: interquartile range; LDH: lactate dehydrogenase; med: median.
[a] For neutrophil count, ESR, procalcytonin, troponin, BNP and LDH levels – highest results were obtained; for lowest results were obtained
*statistically significant ($p < 0.05$)
